# Psychoactive substance disorder: first experience In a comprehensive model of harm reduction model in Bogotá Colombia, 2017-2021

**DOI:** 10.1192/j.eurpsy.2024.818

**Published:** 2024-08-27

**Authors:** A. S. Quesada, M. Cote

**Affiliations:** ^1^Psiquiatría, Universidad de los Andes; ^2^Psiquiatría, Universidad Nacional de Colombia; ^3^EPS Sanitas, Psiquiatra de adicciones de adicciones, Bogotá, Colombia

## Abstract

**Introduction:**

In Colombia the traditional treatment model implies the needing of a total cessation of consume to be able to acces an impatientand long stance rehabilitation program. However, literature in other countries expériences had sugested and used a harm reduction program with an otpatien rehabilitation program.

As this programs are more cost-effective and enables the patient to continue his daily life, perpetuate his life style, keep and enhance the psychosocial network, an outpatient comprehensive multimodular program was designed to adapt to a health promotion company (EPS for its spanish acronym) and has been used since 2017.

**Objectives:**

- share the expérience aquired in an undeveloped country of latin America
- The tipification in the main substance consumption in the development group as well as it’s differentiation in gender and age group

**Methods:**

Expérience and results

**Results:**

- The majority of patients are men over women
- the predominant age group is between 29-59 years old
- there is a difference between the age group depending on the substance of impact

**Conclusions:**

The experience have shown that up to 30% of the population treated have gotten to a controlled consumption or the total suspension without the needing of an impatient program In général thé patient have shown motivation and adherence to an outpatient program

**Disclosure of Interest:**

None Declared

